# Athletic pseudonephritis in male cross-country ultra-marathoners: a comparative observational study

**DOI:** 10.3389/fphys.2025.1608584

**Published:** 2025-08-14

**Authors:** Kai-Hung Chen, Cheng-Hsun Lee, Pang-Yen Chen, Li-Hua Li, Chorng-Kuang How, Yen-Kuang Lin, Yu-Hui Chiu, Feng-Lin Wang, Wei-Fong Kao, Ming-Kun Huang, Ding-Kuo Chien, Wen-Han Chang

**Affiliations:** ^1^ Department of Emergency Medicine, MacKay Memorial Hospital, Taipei, Taiwan; ^2^ Department of Emergency Medicine, Saint Paul’s Hospital, Taoyuan, Taiwan; ^3^ Institute of Biomedical Engineering, National Tsing Hua University, Hsinchu, Taiwan; ^4^ Department of Artificial Intelligence and Medical Application, MacKay Junior College of Medicine, Nursing, and Management, Taipei, Taiwan; ^5^ School of Medicine, College of Medicine, MacKay Medical University, New TaipeiCity, Taiwan; ^6^ Department of Pathology and Laboratory Medicine, Taipei Veterans General Hospital, Taipei, Taiwan; ^7^ Ph.D. Program of Medical Biotechnology, Taipei Medical University, Taipei, Taiwan; ^8^ Department of Emergency Medicine, Taipei Veterans General Hospital, Taipei, Taiwan; ^9^ Department of Emergency Medicine, School of Medicine, National Yang Ming Chiao Tung University, Taipei, Taiwan; ^10^ Graduate Institute of Athletics and Coaching Science, National Taiwan Sport University, Taoyuan, Taiwan; ^11^ Department of Emergency, School of Medicine, College of Medicine, Taipei Medical University, Taipei, Taiwan; ^12^ Department of Electronic Engineering, National Taipei University of Technology, Taipei, Taiwan

**Keywords:** cross-country ultra-marathon, hematuria, myoglobinuria, athletic pseudonephritis, proteinuria, urine cast

## Abstract

**Background/Objective:**

Athletes have commonly reported hematuria, cylindruria, and proteinuria, which are consistent with “athletic pseudonephritis.” To date, little is known about the overall consequences of 100 km (62.5-mile) cross-country ultra-marathons on Asian male runners. This study aimed to examine (i) acute urinary changes in runners with athletic pseudonephritis following a 100 km cross-country ultra-marathon and (ii) whether this phenomenon is associated with the runners average running speed.

**Methods:**

Twenty male Taiwanese ultra-marathoners were prospectively recruited. Urinary parameters were analyzed before and immediately after the 2020 Taiwania 100 K Ultra Trail. Competitors ran on a rugged forest path and the official cut-off time was 15 h.

**Results:**

Participants showed a statistically significant post-race increase (p < 0.001) in urinary red blood cell counts. No significant difference was noted in urinary cast in the immediate post-race values compared to the pre-race values (p = 0.488). Urinary chemistry showed statistically significant increases in specific gravity (p < 0.001), osmolality (p < 0.001), creatinine (p = 0.027), microalbumin (p < 0.001), protein (p < 0.001), and myoglobin (p < 0.001) between the pre- and post-race values. Additionally, 10 (50%) post-race specimens had albumin-to-creatinine ratios >30 mg/g, and 9 (45%) specimens showed protein-to-creatinine ratios >0.2 mg/mg. The association between running speed and red blood cells in urine showed a p-value of 0.368. There were no correlations between running speed and changes in albumin-to-creatinine ratio (rs = −0.105, p = 0.661), protein-to-creatinine ratio (rs = −0.013, p = 0.957), or myoglobin (rs = 0.003, p = 0.99) levels.

**Conclusion:**

Exercise-related hematuria and proteinuria were frequently observed in the Asian male cross-country ultra-marathoners. A faster running speed was not associated with the degree of exercise-induced hematuria, proteinuria or myoglobinuria.

## 1 Introduction

Urinary changes in athletes have become a critical concern due to the increasing number of people engaging in exercise the past few decades (Scheer, 2019). The reported incidence of renal dysfunction during exercise varies widely, ranging from 11% to 100%, depending on the type and intensity of exercise and the athlete’s hydration status (Bellinghieri et al., 2008). Among these changes, athletic pseudonephritis—characterized by elevated urinary erythrocytes, casts, and protein—is the most frequently observed renal dysfunction presentation (Van Biervliet et al., 2013). Given the well-established significance of hematuria and proteinuria as markers of urological and nephrological disorders, their evaluation is essential in cases of evident exercise-induced hematuria.

Prolonged strenuous exercise leads to a decrease in creatinine clearance, resulting in reduced urine flow. Several factors—such as local vascular changes, local hypoxia, lactate accumulation, oxidative stress, hormonal changes, and a general septic reaction—may influence glomerular membrane and tubular cells function in the kidneys during exercise (Shephard, 2016; Wołyniec et al., 2020). These pathophysiological changes result in increased glomerular permeability and decreased renal tubule reabsorption of erythrocytes, casts, and protein (Abarbanel et al., 1990; Fan et al., 2020; Haugen et al., 1980). In addition, myoglobin, a heme-containing protein found in muscle tissues, can contribute to renal stress. During intense or prolonged exercise, the risk of exertional rhabdomyolysis increases due to excessive skeletal muscle breakdown, resulting in myoglobin in circulation and myoglobinuria when the renal threshold is crossed. The overflow of myoglobin can also contribute to proteinuria (Knochel, 1982; Milne, 1988; Rojas-Valverde et al., 2021).

Post-exercise hematuria and proteinuria are usually temporary and can be resolved within a few days without medical supports (Heathcote et al., 2009; Saeed et al., 2012; Varma et al., 2014). However, acute kidney injury (AKI) diagnosis criteria are met in approximately 16%–50% of athletes participating in long-distance races, with endurance runners accounting for 97% of these cases (Rojas-Valverde et al., 2020; Tidmas et al., 2022). The cross-country ultra-marathon (CCUM), which exceeds the classical marathon distance of 42.195 km and takes place on open-air courses over natural terrain, has been growing in popularity. The greater intensity and longer duration of the race results in more evident pathologically and physiologically changes in the competitors’ organs and body systems.

Considering the increasing number of ultra-marathon participants, this observational study aimed to investigate the acute changes in the urinary erythrocytes, casts, and protein in Asian males after a 100 km CCUM race. The secondary aim was to evaluate whether post-exercise hematuria and proteinuria were associated with the participants’ average running speed of these finishers. We believe that these data could provide a practical reference for sports physicians and coaches to make decisions.

## 2 Materials and methods

### 2.1 Study design and population

**FIGURE 1:**
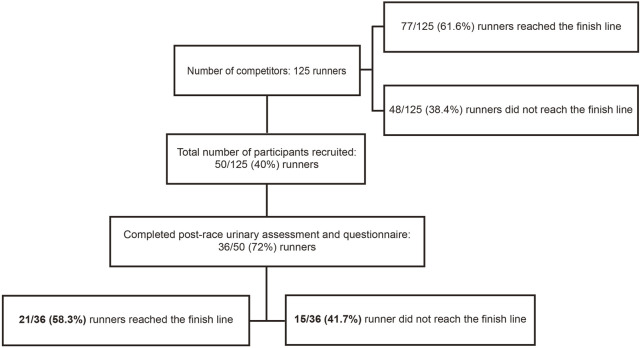
Flow chart of subject recruitment.

Before conducting the observational study, ethical approval was obtained from the MacKay Memorial Hospital Institutional Review Board (20MMHIS114e). Registered runners of the 2020 Taiwania 100 K Ultra Trail were contacted by the study organizer informing them about the study purpose via phone call, 20 days before the race. Fifty competitors volunteered for the study and provided their signed consent before the race. Each of the participants were invited to fill in a questionnaire to provide basic information including their body height, previous marathon or ultra-marathon experience, training protocol for this competition, and their personal best marathon speed. Runners were excluded if they had a history of heart disease, renal dysfunction, seizure, or syncope of unknown origin. Although 36 participants completed the post-race urinary assessment and questionnaire, 15 were excluded from analysis as they failed to complete the race and their urine samples were not collected immediately upon withdrawal. Owing to the gender imbalance, one female finisher was also excluded. Ultimately, the final analysis included data from 20 experienced male Taiwanese ultra-marathoners (Figure 1).

Body weight was measured before and immediately after the race. The competitors were asked to remove the back bag and water bottle that they carried when weighing. All competitors were free to drink any kind of fluid during the race. The mid-stream urine samples were collected before and immediately after the race. The official race records of this 100 K race were obtained to document each participant’s performance.

### 2.2 Laboratory assessment

Urine samples were collected using the clean-catch midstream method to minimize contamination risk. Prior to collection, participants were instructed to cleanse the external genital area with sterile wipes. Urine was collected directly into sterile, clean, dry, and sealed urine tubes compatible with the analytical instruments used. To further reduce contamination, samples were transported to the laboratory within 2 h of collection and analyzed promptly. Samples were assigned non-identifiable codes and examiners were blinded to their origin.

Urinalysis was performed using the Siemens Clinitek Novus Analyzer (Siemens Healthcare, Erlangen, Germany), a fully automated system that integrates both urine chemistry and sediment analysis. The chemistry analyzer utilizes high-resolution digital imaging to interpret dipstick tests, providing semi-quantitative measurements for parameters including pH, red blood cell (RBC) counts, and specific gravity (SG). For sediment analysis, the system employs automated digital microscopy in combination with image recognition software to detect and classify urinary casts. When hematuria is suspected, the urine sample is centrifuged and examined microscopically to identify RBCs and other sediment components. A manual review by trained personnel is performed when results are flagged, ambiguous, or clinically significant, ensuring accurate interpretation and reporting. In accordance with the 2020 American Urological Association Guideline on Microhematuria, hematuria in this study was defined as ≥3 RBCs per high-power field (HPF) in microscopic urine samples (Barocas et al., 2020).

Urinary osmolality was determined using the freezing point depression method using the Advanced Model 3,320 Micro-Osmometer (Advanced Instruments, Norwood, MA, United States). Urinary parameters including creatinine, microalbumin, myoglobin and protein were analyzed using the Beckman Coulter AU5800 Clinical Chemistry Analyzer (Beckman Coulter Inc., Brea CA, United States).

### 2.3 Track briefing and weather condition

The race was held on 19 September 2020, in one of the largest divine-tree gardens in Asia in Yilan, Taiwan (24° 35′N latitude and 121° 26′E longitude). The race was officially announced by the International Trail Running Association (ITRA) as a graded race. Competitors ran a total of 100 km on a rugged forest path, with an overall cut-off time of 15 h. The race was conducted on a sunny day with temperatures ranging between 15.4°C and 32.8°C. The relative humidity was between 62% and 100%, and the wind speed was between 0 m/s and 1.5 m/s, according to data provided by the Central Weather Bureau, Taiwan.

### 2.4 Statistical analysis

The Shapiro-Wilk test (p > 0.05) and an inspection of the participants’ histograms, normal Q-Q plots, and box plots were used to test the normality of the data. Descriptive results are reported as median (interquartile range (IQR)). The Fisher exact probability test was applied for categorical data and dependent-samples t-test (or Wilcoxon Signed Ranks test, when appropriate) for numerical data to evaluate the physiological and biochemical association between pre- and post-race parameters. Multiple comparisons were controlled using the Bonferroni correction. The Kruskal–Wallis test was applied for evaluating the association between running speed and urine RBCs/HPF. The Spearman’s rank correlation coefficient was used to evaluate the association between running speed and delta changes (i.e., post-race value - pre-race value) in body weights and urinary biochemistry. A commercially available statistical software (SPSS version 21.0, IBM Corp., Armonk, NY, United States) was used for statistical analysis. Differences were statistically significant when the two-tailed p-value was <0.05.

## 3 Results

### 3.1 Race details and runner demographics

A total of 125 runners participated in this race. The median age of the runners was 46.5 years (IQR, 12 years). Furthermore, 110/125 (88%) runners were male, 77 (61.6%) finished the race, and 71 of the 77 finishers (92.2%) were male. The average finishing time was 13.6 h (range, 10–14.9 h). Only 50 (40%) competitors volunteered to participate in this study and completed the pre-race urinary assessment and questionnaire. Of these, 36 participants (33 males and 3 females) completed the post-race urinary assessment and questionnaire, and of these 21 (20 males and 1 female) finished the race (Figure 1). The characteristics of the included 20 male finishers are summarized in Table 1.

**TABLE 1:** Demographic data and outcome measurement of participants (n = 20).

Variable	Median (IQR)
Age (years)	46.5 (12)
Pre-race BW (kg)	69.9 (5.3)
Height (m)	172 (7)
Body mass index (kg/m^2^)	23.7 (2.1)
Years of running marathon	7 (5.8)
Years of running ultra-marathon	5 (6)
Training protocol (/week)	
<40 km	1
40–100 km	10
>100 km	9
Best marathon time (min)	209.5 (34)
Average running speed in this race (km/hr)	7.09 (0.62)

IQR, interquartile range; BW, body weight. Kg = kilogram; m = meter; min = minute; hr = hour.

### 3.2 Analysis of urinary parameters

**FIGURE 2:**
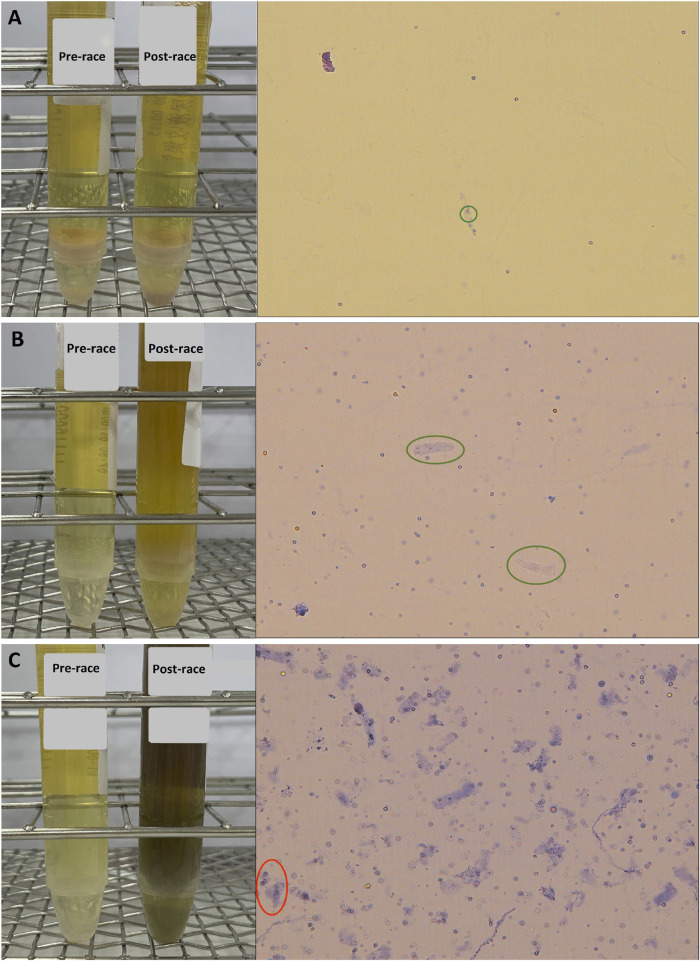
Pre- and post-race urinalyses with microscopic photographs of urine casts observed following the 100 km cross-country ultra-marathon. **(A)** Hyaline cast observed at a count of 0–5 per low-power field (LPF) (green circle); **(B)** Hyaline casts increased to 5–10 per LPF (green circle); **(C)** Numerous hyaline casts observed at >25 per LPF, along with granular casts at 2–5 per LPF (red circle).

Urinalysis of the finishers’s specimens were measured at two time points: before the race and immediately after the race. Comparison of the urine specimens’ RBCs and casts are shown in Table 2. No RBC counts above the reference interval (≥3 cells/HPF) were observed in the 20 participants pre-race. Post-race, twelve (60%) finishers presented with hematuria (RBC counts ≥3 cells/HPF). The difference between pre- and post-race urine RBC was statistically significant (p < 0.001). Regarding urine cast, 2 (10%) finishers presented with hyaline cast >5 cells per low-power field (LPF) after the run. There was no significant difference in the immediate post-race values compared to the pre-race values (p = 0.488). Figure 2 illustrates the pre- and post-race urinalyses results, demonstrating the microscopic images of urine casts observed in the samples.

**TABLE 2:** Red blood cells (RBCs) and hyaline casts in urine (n = 20).

Parameter	Pre-race	Post-race	p value
RBCs/HPF			
0–2	20	8	**<0.001**
3–50	0	4	
51–100	0	4	
>100	0	4	
Hyaline Casts/LPF			
0–5	20	18	0.488
6–10	0	1	
11–25	0	0	
>25	0	1	

Urine chemistry of these finishers’ specimens were also measured at the two time points: before the race and immediately after the race. The exact measurement values are listed in Table 3. An increase in SG, osmolality, creatinine, microalbumin, albumin-to-creatinine ratio (ACR), protein, protein-to-creatinine ratio (PCR), and myoglobin showed significant differences (p < 0.05) in the immediate post-race values compared to the pre-race values. After the run, 10 (50%) finishers presented ACR values greater than 30 mg/g. In addition, 9 (45%) finishers exhibited PCR values greater than 0.2 mg/mg in post-race specimens.

**TABLE 3:** Urine chemistries before and after the ultra-marathon (n = 20).

Parameter	Median (IQR)			
	Pre-race	Post-race	p-value	Effect size
PH	6 (0.9)	5.5 (1.4)	1	−0.10
Specific Gravity^a^	1.02 (0.006)	1.028 (0.01)	**<0.001**	**1.33**
Osmolality (mOsm/kg)^a^	706 (284)	932.5 (206)	**<0.001**	**1.40**
Creatinine (mg/dL)^a^	119.2 (54.1)	160.1 (96)	**0.027**	**0.90**
Microalbumin (mg/dL)	0 (0.9)	6.3 (21.7)	**<0.001**	**0.59**
ACR (mg/g)	0 (5.8)	31.8 (98.2)	**<0.001**	**0.6**
Protein (mg/dL)	8.5 (6)	32 (57)	**<0.001**	**0.61**
PCR (mg/g)	72 (29)	189.5 (216)	**<0.001**	**0.61**
Myoglobin (ng/mL)	0 (0)	16 (50.9)	**<0.001**	**0.57**

vs = versus; IQR, interquartile range; mg/dL = milligrams per deciliter.

ACR, albumin to creatinine ratio; mg/g = milligrams per Gram.

PCR, protein to creatinine ratio; ng/mL = nanograms per milliliter.

^a^ It follows normal distribution; p-values were obtained from parametric methods.

### 3.3 Correlation analyses between running speed and urinary parameters

**FIGURE 3:**
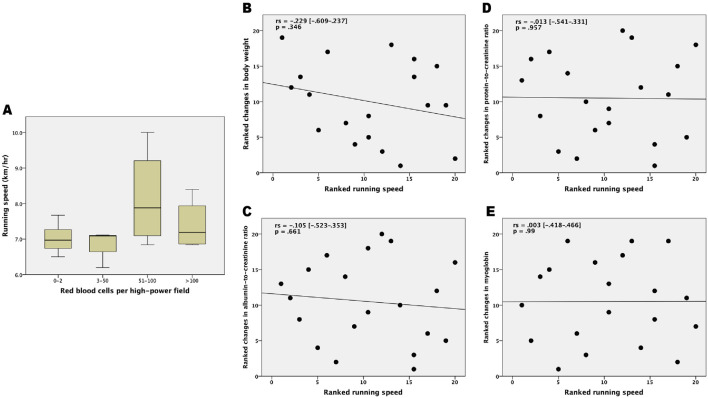
Correlation between running speed and measured parameters following the 100 km cross-country ultra-marathon. **(A)** Box plot for comparing the running speed across various categories of urine red blood cells; **(B)** Ranked running speed versus ranked delta changes in body weight; **(C)** Ranked running speed versus ranked delta changes in albumin-to-creatinine ratio; **(D)** Ranked running speed versus ranked delta changes in protein-to-creatinine ratio; and **(E)** Ranked running speed versus ranked delta changes in myoglobin. rs = Spearman rank correlation coefficient.

The association between running speed and urine RBCs/HPF in immediately following species are shown in Figure 3A. The significance was 0.368. No correlations were observed between running speed and changes in body weights (rs = −0.229 [−0.609–0.237], p = 0.346), and the ACR (rs = −0.105 [−0.523–0.353], p = 0.661), PCR (rs = −0.013 [−0.541–0.331], p = 0.957), or myoglobin (rs = 0.003 [−0.418–0.466], p = 0.99) levels. The correlation between running speed with delta changes in body weights and urinary biochemistry is shown graphically in Figures 3B–E with ranked variables.

## 4 Discussion

Exercise-induced hematuria is common among individuals who participate in a variety of sport categories. Although self-limiting, the repeated erythrocytes loss through the urine can be a contributing factor to the anemia (Abarbanel et al., 1990; Dang, 2001). The incidence of hematuria in ultra-marathoners following 90–100 km races are reported to range from 12% to 54%, with studies indicating that it typically resolves within a few hours to 7 days (Alvarez et al., 1987; Chiu et al., 2015; Kallmeyer and Miller, 1993; Varma et al., 2014). The 60% incidence of hematuria in finishers included in the present study differs from the 24.4% reported in Kallmeyer’s recruits (Kallmeyer and Miller, 1993), but it is similar to the 53.8% observed by Alvarez (Alvarez et al., 1987). Exercise-induced hematuria is influenced by both the duration and intensity of exercise (Bellinghieri et al., 2008; McInnis et al., 1998). However, no association between running speed and hematuria was observed in the present study.

As for cylindruria, hyaline casts can be found in normal cases of paraphysiological conditions such as after strenuous exercise, and in non-renal disorders such as dehydration. During intense physical activity, renal blood flow can decrease markedly—from approximately 1,000 mL/min at rest to as low as 200 mL/min (Bellinghieri et al., 2008). In addition, a reduction in urine output below 25 mL/h was associated with a significant increase in urinary casts (Haugen et al., 1980). Notably, 2/20 (10%) finishers presented hyaline casts after the race. One of the finishers who exhibited hyaline casts >25/LPF also exhibited granular casts. The presence of granular casts is commonly associated with renal tubular damage (Caleffi and Lippi, 2015; Chawla et al., 2008). Taken together, these results might imply that acute adverse effects on glomerular function occur in response to long-distance running.

Morteza-Khodaee’s study showed that 30% of runners had proteinuria (≥1+) after completing a 161-km ultra-marathon (Khodaee et al., 2021). In the present study, 45% of the included finishers exhibited proteinuria (spot urine PCR >0.2 mg/mg), based on the most reported cutoff for detecting proteinuria (Kamińska et al., 2020). Albuminuria is an early and sensitive marker of kidney risk (de Zeeuw, 2004) and albumin is the main protein lost during exercise (Heathcote et al., 2009; Poortmans et al., 1989). After running, albuminuria can reach a 10 to 25-fold increase in healthy individuals (Poortmans et al., 1997; Poortmans et al., 1989). Wołyniec and colleagues have reported that ACR increased from 5.37 to 49.64 mg/g after a 100 km ultra-marathon (Wołyniec et al., 2020). The ACR of the finishers included in the present study increased from 0 to 31.8 mg/g after the 100 km cross-country run. This data, although far from conclusive, provides additional insights into urine ACR and PCR—important biomarkers of kidney damage—in long-distance running sports.

In the literature discussing during very long runs, the post-exercise proteinuria is said to be related to the duration but not the intensity of exercise (Wołyniec et al., 2020). The data of the present study showed no correlation between running speed and the degree of proteinuria and myoglobinuria. Notably, 13 (65%) of the finishers observed in the present study presented with urinary myoglobin levels above 5 ng/mL after the run. The wide variability in post-race myoglobin levels suggests inter-individual differences that may be influenced by factors such as hydration status, muscle mass, or baseline conditioning. However, myoglobinuria may be asymptomatic, transient, and reversible, and visible myoglobinuria (tea colored or brown colored urine) only occurs when urinary myoglobin exceeds 250 μg/mL (Scalco et al., 2016; Warren et al., 2002). Ultra-marathoners frequently compete in endurance events, where repeated myoglobin overflow may contribute to renal injury through renal vasoconstriction, proximal tubular necrosis, and distal tubular obstruction (Bosch et al., 2009).

As measurements during CCUMs are challenging, the time course of urinary data during ultra-endurance is limited. The strength of our study is that it presents acute urinary changes among finishers of a 100 km CCUM event. The present study has some limitations. First, the small sample size resulted in decreased power to detect significant post-race changes in the evaluated parameters. As CCUM is an extreme sport, it is difficult to enroll many individuals. Second, only male runners were included in the final analysis, which may account for sex differences in the urine data. Therefore, the data presented might not be representative of potential urinary parameter changes in female runners. Third, follow-up urinary tests on participants beyond the immediately post-race period were not performed. Hence, future studies measuring the long-term effects of CCUM races are necessary.

To date, no prior literature has explored acute urinary changes of athletic pseudonephritis in Asian ultra-marathoners during a CCUM. Although our study focuses on immediate post-race changes, it highlights important questions about the potential long-term consequences of repeated episodes of exertional hematuria and proteinuria. Incorporating longitudinal studies and post-race follow-ups would position this work within a broader clinical and research context. Such future investigations could shed light on whether these acute urinary changes fully resolve or contribute to cumulative renal stress over time. We anticipate that further data on ultra-marathons could provide additional insights into this issue.

### 4.1 Conclusion

Athletic pseudonephritis was observed in participants after a 100 km CCUM. The development of exercise-related hematuria and proteinuria was frequently observed among these runners. A faster average running speed had no significant effect on exercise-induced hematuria, proteinuria or myoglobinuria in the included finishers. These results are potentially relevant to coaches, athletic trainers, and race organizers, who might apply them to hydration planning, post-race screening, or athlete education.

## Data Availability

The original contributions presented in the study are included in the article/supplementary material, further inquiries can be directed to the corresponding author.
